# Supplementary material for: Functional requirements of the liver isoform of phosphofructokinase-1 in breast cancer cell migration
Source: J Cell Sci. 2025 Dec 4;138(23):jcs264251. doi: 10.1242/jcs.264251 (PMC12752486; doi:10.1242/jcs.264251)
Supplement: Supplementary information [file joces-138-264251-s1.pdf]

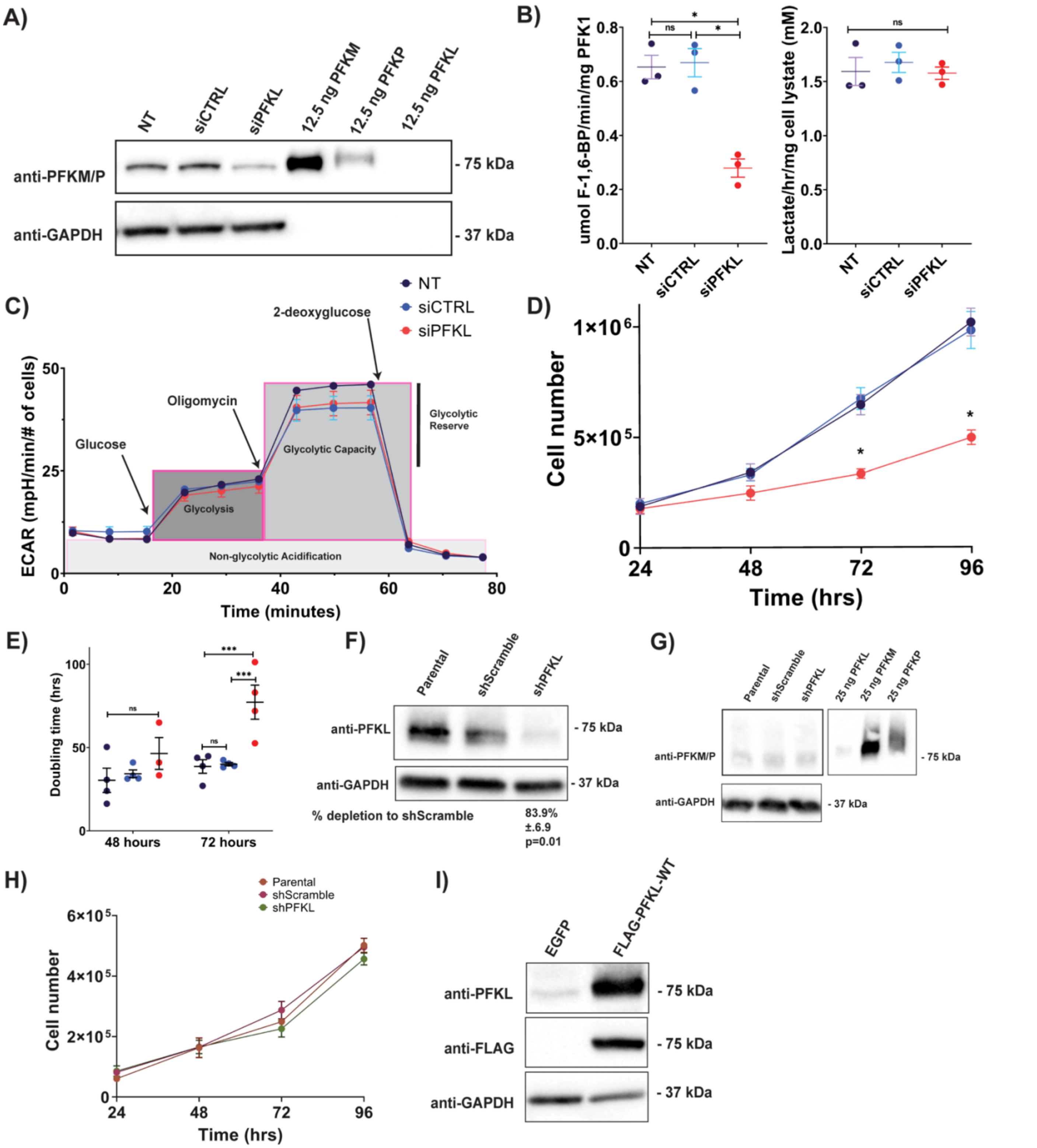

Fig. S1. Characterization of siRNA and shRNA PFKL depletion in MDA-MB-231 cells.

A) Western blot of cell lysates of non-transfected (NT), control siRNA transfected (siCTRL), or PFKL siRNA (siPFKL) transfected cells probed with antibodies raised against PFKM/P or GAPDH as a loading control. 12.5 ng of recombinant protein used as an antibody control. **B)** Total PFK1 activity out of cell lysates of NT, siCTRL, and siPFKL transfected cells 72 hours post-transfection, confirming depletion of PFKL by siRNA. *P<0.05 calculated by ordinary one-way ANOVA followed by Turkey's multiple comparison test, n=3. Error

bars represent SEM (Right). Lactic acid efflux measured from intact NT, siCTRL, and siPFKL cells 72 hours post siRNA transfection. No significant changes in lactic acid flux were observed (Left). **C)** ECAR trace of NT, siCTRL, and siPFKL transfected cells. Arrows indicate addition of glucose, oligomycin, and 2-deoxyglucose. Glycolysis indicates a measure of basal glycolytic flux upon addition of glucose (dark grey box). Glycolytic capacity indicates the maximum glycolytic rate that can be achieved upon mitochondrial inhibition with oligomycin (medium grey box). Non-glycolytic acidification indicates lactate production from other non-glycolytic pathways in the cells (light grey box). Glycolytic reserve indicates the difference between maximum glycolytic rate (and basal glycolysis (black labeled line). **D)** Proliferation curve of NT, siCTRL, and siPFKL transfected cells 24 to 96 hours post-transfection, n=3 (left). There is a significant decrease in cell count at 72 and 96 hours. **E)** Doubling times calculated for 48 hours and 72 hours post-transfection (right). ***P<0.001 calculated by ordinary one-way ANOVA followed by Turkey's multiple comparison test. Error bars represent SEM, n=3. **F)** Western blot of lysates from MDA-MB-231 parental cells, and cells transduced shScramble or shPFKL, probed with antibodies raised against PFKL and GAPDH (right). Percent of PFKL expression of shScramble and shPFKL listed below western blot. n=3 biological replicates, significance determined using paired t-test. PFKM/P or GAPDH (left). 25 ng recombinant protein of PFKL, PFKP, and PFKM loaded as antibody control for PFKM/P blot (Spliced to reduce spacing between sample groups). **H)** Proliferation curve of MDA-MB-231 parental and cells transduced with shScramble or shPFKL between 24 and 96 hours, n=3 biological replicates. ns, not significant between all sample groups. **I)** Western blot of MDA-MB-231 cells lysate transduced with EGFP or FLAG-PFKL-WT, probed with antibodies raised against PFKL, FLAG M2, or GAPDH.

Journal of Cell Science • Supplementary information

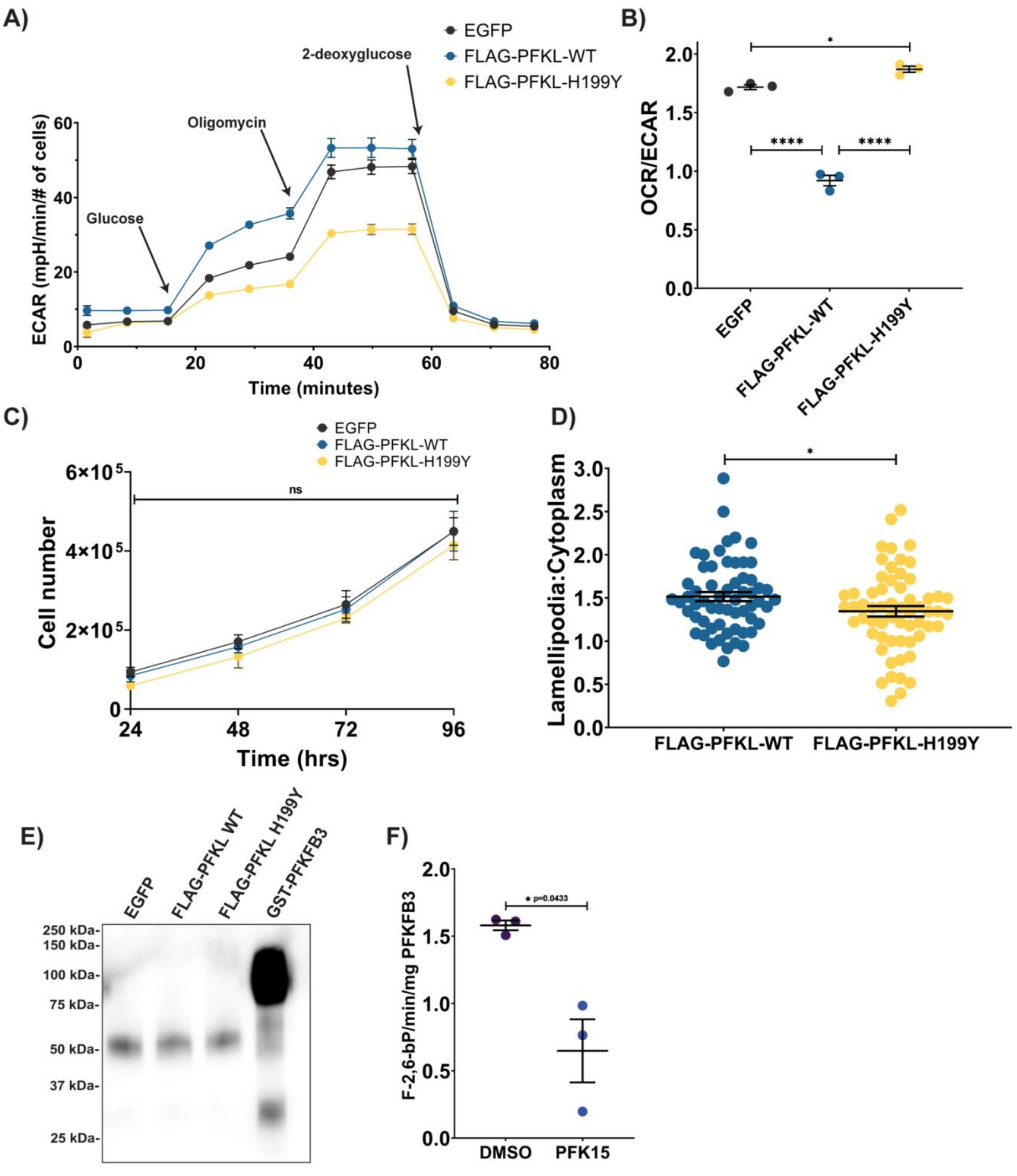

Journal of Cell Science • Supplementary information

Fig. S2. Characterization of cells expressing catalytically inactive PFKL A) ECAR trace of EGFP, FLAG-PFKL- WT, and FLAG-PFKL-H199Y transduced cells. Arrows indicate addition of glucose, oligomycin, and 2 -deoxyglucose. **B)** OCR-to-ECAR ratio of different cell lines. FLAG-PFKL-WT has significant decrease in ration due to increased glycolysis. Data are means of three independent experiments, and error bars represent SEM. ****P<0.0001 determined by ordinary one-way ANOVA. **C)** Proliferation curve of MDA-MB-231 cells transduced with EGFP, FLAG-PFKL-WT, and FLAG-PFKL-H199Y between 24 and 96 hours, n=3. NS, not significant between all sample groups. D) Quantification of 60 representative FLAG-PFKL-WT and FLAG-PFKL-H199Y cells from four independent experiments using line scans spanning lamellipodia and cytoplasm. Significance calculated using paired t-test (p=0.04) E) Western blot of MDA-MD- 231 cells probed with antibody raised against PFKFB3 (~50 kDa). 6.25ng of recombinant GST-tagged PFKFB3 (~87 kDa) used as antibody control. F) Kinetic plate reader assay measuring activity of 25 ng recombinant GST -PFKFB3 treated with DMSO or 2μM PFK15. *P<0.05 calculated with paired t-test. Bars represent SEM, n=3.

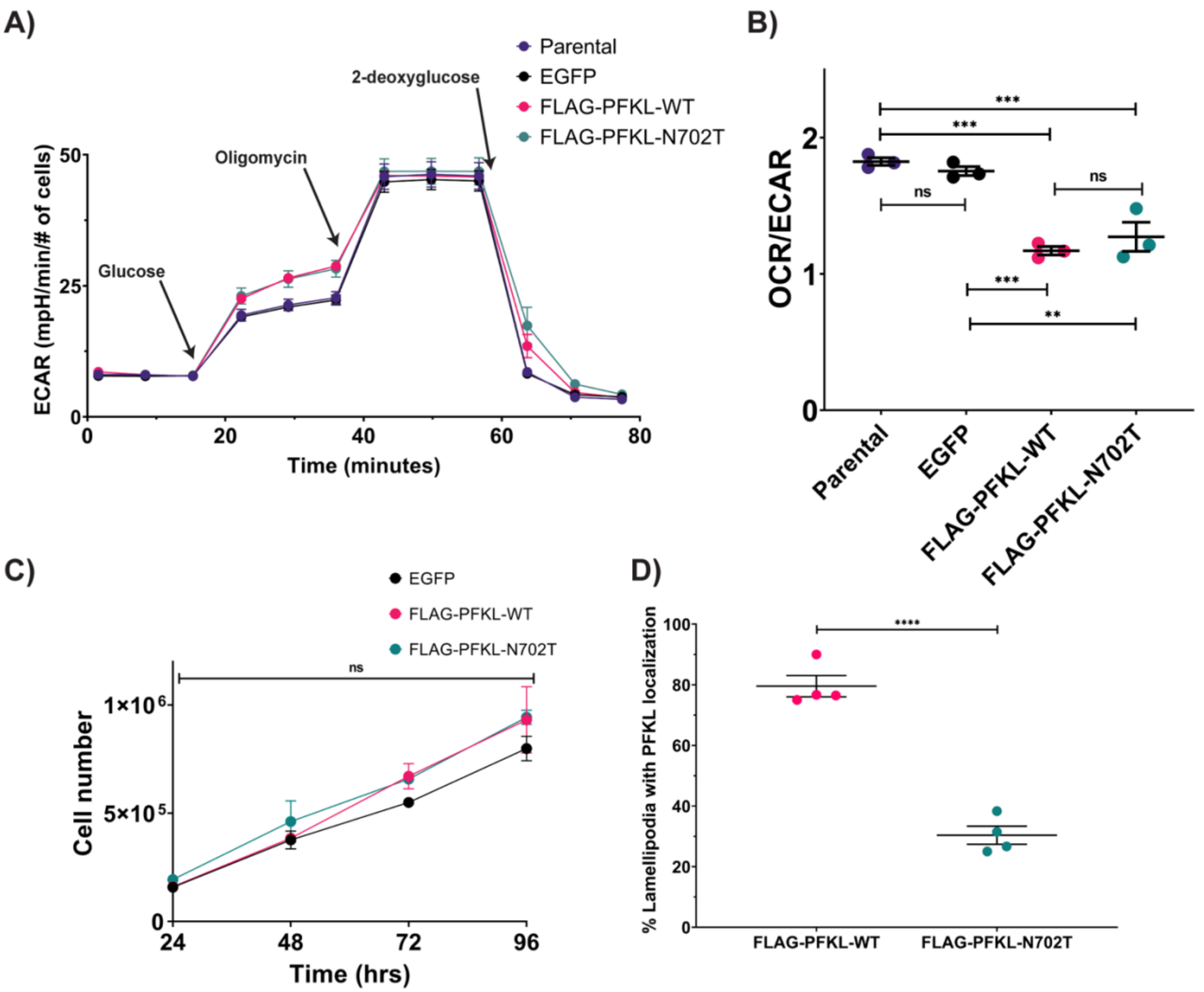

Fig. S3. Characterization of cells expressing filament incompetent PFKL-N702T. A) ECAR trace of EGFP, FLAG-PFKL- WT, and FLAG-PFKL-N702T transduced cells. Arrows indicate addition of glucose, oligomycin, and 2-deoxyglucose. **B)** OCR-to-ECAR ratio of different cell lines. FLAG-PFKL-WT and PFKL-N702T have significant decreases in ratio due to increased glycolysis. However, there is no significant difference between the two. Data are means of three independent experiments, and error bars represent SEM. *P<0.05 determined by ordinary one-way ANOVA. **C)** Proliferation curve of EGFP, FLAG-PFKL-WT, and FLAG-PFKL-N702T between 24 and 96 hours, n=3. NS, not significant between all sample groups. **D)** Quantification of FLAG-PFKL-WT or FLAG-PFKL-N702T expressing cells with PFKL localization to lamellipodia. n=4 biological replicates. 124 FLAG-PFKL-WT and 109 FLAG-PFKL-N702T cells were quantified. Significance was determined by paired t-test (p= 4.9E-5).

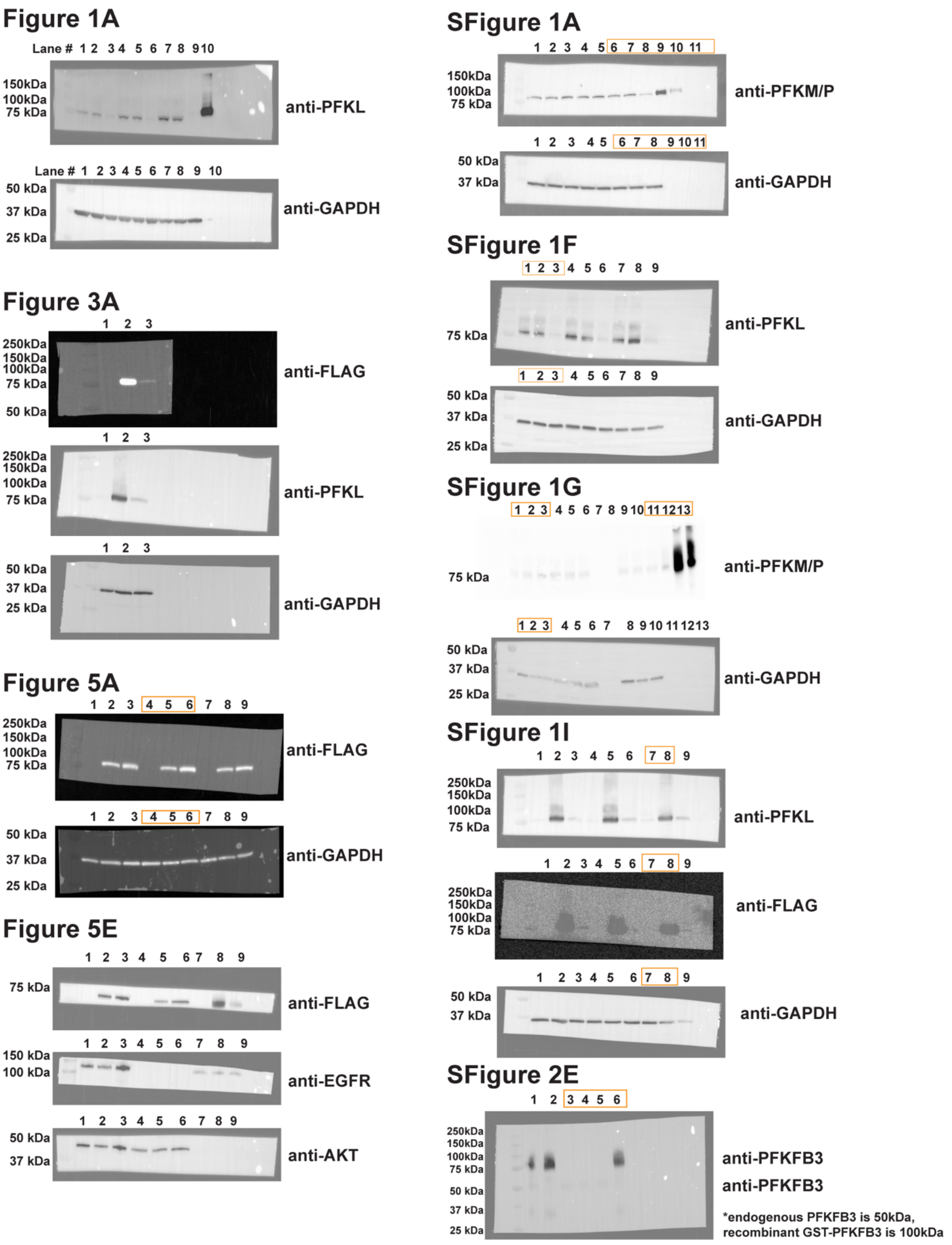

Fig. S4. Blot transparency. Original uncropped blots labeled for which figure they are located in. All loaded lanes are labeled, and the antibody used as well as molecular weight of protein of interest is noted next to each blot. Orange boxes indicate which lanes were chosen to use in figures. If no lanes are boxed, this indicates the whole blot was used.

Journal of Cell Science • Supplementary information

Table S1. Migration parameters of MDA-MB-231 non-transfected, siRNA control transfected (siCTRL), and PFKL siRNA transfected (siPFKL).

Treatment	Non-transfected	siControl	siPFKL
Cell Number	104	104	105
Velocity (µm/min)	0.61 ±0.22	0.52 ±0.21	0.39 ±0.15
Euclidean distance (µm)	110.47 ±48.75	98.34 ±46.29	65.37 ±41.64
Accumulated distance (µm)	216.75 ±78.10	187.28 ±78.75	171.83 ±78.41
Directionality (euc/accum)	0.53	0.54	0.42
X Forward Migration Index (X-FMI)	-0.4	-0.35	-0.03
Y Forward Migration Index (Y-FMI)	0.01	-0.01	-0.04
Rayleigh p-value	3.06E-24	2.06E-19	0.052

Table S2. Migration parameters of Parental MDA-MB-231 cells, or cells transduced with shScramble or shPFKL.

Cell Type	Parental	shScramble	shPFKL
Cell Number	100	140	145
Velocity (µm/min)	0.63 ±0.28	0.66 ±0.37	0.41 ±0.21
Euclidean distance (µm)	233.18 ±100.46	202.57 ±119.32	124.5 ±73.07
Accumulated distance (µm)	447.79 ±169.01	390.59 ±166.76	246.33 ±106.39
Directionality (euc/accum)	0.55	0.53	0.53
X Forward Migration Index (X-FMI)	-0.45	-0.38	-0.11
Y Forward Migration Index (Y-FMI)	0.05	-0.07	-0.02
Rayleigh p-value	2.49E-29	1.18E-28	0.035

Table S3. Migration parameters of MDA-MB-231 cells transduced with EGFP, FLAG- PFKL-WT, or FLAG-PFKL-H199Y.

Cell Type	EGFP	FLAG-PFKL	FLAG-PFKL-H199Y
Cell Number	91	88	111
Velocity (µm/min)	0.59 +- 0.15	0.59 +- 0.12	0.53 +-0.14
Euclidean distance (µm)	256.94 +- 95.73	251.4+-97.48	153.64+-77.05
Accumulated distance (µm)	538.78+-142.02	525.41+-125.01	475.39+-124.12
Directionality (euc/accum)	0.49	0.49	0.34
X Forward Migration Index (X-FMI)	-0.39	-0.36	-0.05
Y Forward Migration Index (Y-FMI)	-0.05	-0.07	0.06
Rayleigh p-value	3.13E-24	1.38E-20	0.01

Table S4. Migration parameters for MDA-MB-231 cells treated with DMSO or 2 μM PFK15.

Treatment	DMSO	PFK15
Cell Number	130	135
Velocity (μm/min)	0.53+-0.14	0.31+-0.12
Euclidean distance (μm)	212.95+-78.75	116.38+-66.05
Accumulated distance (μm)	500.616+-137.4	301.92+-111.69
Directionality (euc/accum)	0.44	0.39
X Forward Migration Index (X-FMI)	-0.34	-0.22
Y Forward Migration Index (Y-FMI)	-0.07	0.05
Rayleigh p-value	6.73E-35	1.28E-16

Table S5. Migration parameters of MDA-MB-231 transduced with EGFP, FLAG- PFKL-WT, or FLAG-PFKL-N702T.

Cell type	EGFP	FLAG-PFKL	FLAG-PFKL-N702T
Cell Number	150	150	150
Velocity (μm/min)	0.5 ±0.15	0.48 ±0.15	0.42 ±0.16
Euclidean distance (μm)	128.26 ±60.69	118.66 ±60.26	92.8 ±45.32
Accumulated distance (μm)	241.95 ±86.83	219.91 ±85.75	212.18 ±97.82
Directionality (euc/accum)	0.55	0.57	0.48
X Forward Migration Index (X-FMI)	-0.44	-0.37	-0.08
Y Forward Migration Index (Y-FMI)	-0.04	0	0.04
Rayleigh p-value	6.78E-25	1.81E-17	0.004

Journal of Cell Science • Supplementary information
